# Effect of Oocyte Quality Assessed by Brilliant Cresyl Blue (BCB) Staining on Cumulus Cell Expansion and Sonic Hedgehog Signaling in Porcine during In Vitro Maturation

**DOI:** 10.3390/ijms21124423

**Published:** 2020-06-22

**Authors:** Sanghoon Lee, Hyo-Gu Kang, Pil-Soo Jeong, Tsevelmaa Nanjidsuren, Bong-Seok Song, Yeung Bae Jin, Sang-Rae Lee, Sun-Uk Kim, Bo-Woong Sim

**Affiliations:** 1 Futuristic Animal Resource & Research Center, Korea Research Institute of Bioscience and Biotechnology, Chungcheongbuk-do 28116, Korea; sodany2@kribb.re.kr (S.L.); kogd1887@kribb.re.kr (H.-G.K.); spectrum@kribb.re.kr (P.-S.J.); tsevelmaa@kribb.re.kr (T.N.); sbs6401@kribb.re.kr (B.-S.S.); sunuk@kribb.re.kr (S.-U.K.); 2 National Primate Research Center, Korea Research Institute of Bioscience and Biotechnology, Chungcheongbuk-do 28116, Korea; ybjin@kribb.re.kr (Y.B.J.); srlee@kribb.re.kr (S.-R.L.); 3 Department of Functional Genomics, KRIBB School of Bioscience, Korea University of Science and Technology, Daejeon 34113, Korea

**Keywords:** oocyte quality, brilliant cresyl blue, cumulus cell expansion, Sonic hedgehog signaling, apoptosis

## Abstract

Brilliant cresyl blue (BCB) staining is used to select developmentally competent cumulus-oocyte complexes (COCs) for in vitro maturation (IVM). However, limited attention has been paid to what drives the higher developmental competence of BCB+ COCs. Sonic hedgehog signaling (SHH) is an important signaling pathway for ovarian follicular development and oocyte maturation. Therefore, this study investigated the effect of oocyte quality assessed by BCB staining on cumulus cell expansion, oocyte nuclear maturation, subsequent embryo development, apoptosis levels, and SHH signaling protein expression, in porcine COCs. After IVM, BCB+ COCs exhibited a significantly higher proportion of complete cumulus cell expansion and metaphase II rate in oocytes than BCB- COCs. After in vitro fertilization, the BCB+ group showed a significantly higher monospermy rate, fertilization efficiency, percentage of cleavage and blastocyst formation, with a higher total cell number and a lower apoptosis in blastocysts as compared with the BCB- group. Furthermore, significantly lower apoptosis levels and a higher expression of SHH-signaling proteins in COCs were observed, before and after IVM. In conclusion, high-quality oocytes had a greater potential to expand their surrounding cumulus cells with active SHH signaling and a lower apoptosis. This could provide COCs with a proper environment for maturation, thereby leading to a better subsequent embryo development.

## 1. Introduction

The in vitro maturation (IVM) of immature oocytes is an important assisted reproductive technology (ART) capable of generating mature oocytes that can develop to term [1]. Despite the impressive success in the application of IVM, only 30–35% of immature mammalian oocytes develop to the blastocyst stage in vitro, after oocyte maturation, insemination, and embryo culturing [2,3,4]. One of the reasons for this low developmental competence is thought to be low-quality oocytes submitted to IVM. As the recurrent failure in ART could be explained by low-quality oocytes causing a lack of sperm penetration, an oocyte activation failure, and a blockage of embryo development, selecting high-quality oocytes is crucial for improving ART [4,5,6].

Brilliant cresyl blue (BCB) has been used to select more competent oocytes prior to IVM in various species, including pigs, mice, goats, cattle, and buffaloes [7]. In 1993, Ericsson et al. suggested, for the first time, a BCB test to select porcine oocytes with a higher developmental competence [8]. The staining allows for the determination of the glucose-6-phosphate dehydrogenase (G6PDH) activity, which converts BCB dye from blue to colorless [9]. The oocytes, which are still in the growth phase, have a high G6PDH activity and show a colorless cytoplasm (BCB-). However, oocytes that have completed their growth have low levels of G6PDH and show a blue coloration of the cytoplasm (BCB+). BCB+ oocytes have a significantly higher blastocyst developmental rate than BCB- oocytes, suggesting that the quality of BCB+ oocytes is higher than that of BCB- oocytes [4]. While many studies have demonstrated that BCB+ oocytes have a higher developmental competence as compared with BCB- oocytes, little information is available on why a higher percentage of BCB+ oocytes develop to the blastocyst stage. It has been suggested that higher rates of subsequent embryo development of oocytes can be attributed to cumulus cell expansion [10], and the relationship between cumulus cell expansion and the quality of oocytes could be regulated by Sonic hedgehog (SHH) signaling [11,12].

Cumulus cell expansion is an essential condition for oocyte maturation and the acquisition of developmental competence [13]. A sufficient number of cumulus cell layers and adequate hyaluronic acid (HA) production, followed by cumulus cell expansion, are necessary for successful oocyte maturation and subsequent embryo development [14,15,16]. In contrast, an insufficient binding of the HA to the CD44 receptor during the IVM of COCs leads to an unsuccessful oocyte maturation and subsequent embryo development [17].

SHH signaling is involved in the development of the ovary [18] and is active in the granulosa cells surrounding the follicle [19]. In growing ovarian follicles, the oocyte and surrounding somatic cells regulate each other’s proliferation and differentiation by communicating via signaling pathways [20], such as SHH, which is essential for development and patterning in many tissues [19]. When the SHH ligand binds to the Patched1 (PTCH1) receptor on the cell surface, its inhibitory action on Smoothened (SMO), the seven-transmembrane G-protein-coupled coreceptor, is relieved. This allows SMO to activate the glioma-associated oncogene homolog 1 (GLI1) transcription factor, resulting in an upregulation of the target genes, which control cell patterning, proliferation, and differentiation during development [21]. Recently, these elements (PTCH1, SMO, and GLI1) of active SHH signaling were found in the granulosa and cumulus cell layer of oocytes, and supplementation with SHH protein during the IVM of oocytes was demonstrated to promote oocyte maturation and subsequent embryo development [22].

Considering the interactive cellular milieu in which oocytes grow and the role of surrounding somatic cells, such as cumulus cells, it was thought that differential levels of cumulus cell expansion and SHH signaling would be observed between BCB- and BCB+ cumulus-oocyte complexes (COCs), after IVM. Therefore, we aimed to determine whether BCB+ COCs have more potential to expand cumulus cells with active SHH signaling to provide COCs with a proper environment for maturation. In this study, pig oocytes were used as a model of low-quality (BCB-) and high-quality (BCB+) oocytes. We investigated the cumulus cell expansion, nuclear maturation of oocytes, subsequent development of parthenogenetic activation (PA) and in vitro fertilization (IVF) embryos, apoptosis levels, and SHH signaling in COCs between BCB- and BCB+ porcine COCs.

## 2. Results

### 2.1. Evaluation of the Cumulus Cell Expansion and Nuclear Maturation of Brilliant Cresyl Blue (BCB-) and BCB+ COCs

**Figure 1:**
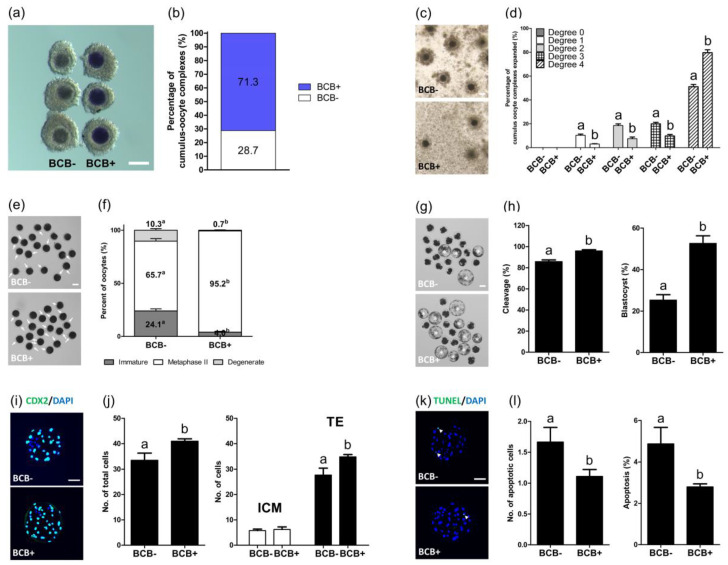
Comparison of brilliant cresyl blue (BCB-) and BCB+ cumulus-oocyte complexes (COCs) in terms of cumulus expansion, nuclear maturation, and subsequent embryo development after parthenogenetic activation. (**a**) Representative photomicrographs of germinal vesicle-stage porcine COCs after BCB staining. The colorless ooplasm indicates BCB- COCs, and the blue ooplasm indicates BCB+ COCs. (**b**) The ratio of BCB- to BCB+ COCs after staining. (**c**) Representative photomicrographs, and (**d**) the percentage of the degree of the cumulus expansion of BCB- and BCB+ COCs, after 44 h of IVM. (**e**) Representative photomicrograph of in vitro matured oocytes, and (**f**) the percentage of different stages of nuclear maturation in BCB- and BCB+ oocytes, after 44 h of IVM. The white arrows indicate in vitro matured oocytes with a first polar body. (**g**) Representative photomicrographs of blastocysts (white asterisks), and (**h**) the cleavage and blastocyst formation rates of embryos developed from BCB- and BCB+ oocytes. (**i**) Immunocytochemistry of CDX2/4′,6-diamidino-2-phenylindole (DAPI) using blastocysts developed from the indicated groups. Merged images of CDX2 (green) and DAPI (blue) signals are shown. (**j**) Quantification of the total, inner cell mass (ICM), and trophectoderm (TE) cell numbers in the indicated groups. (**k**) Terminal deoxynucleotidyl transferase mediated dUTP digoxygenin nick end labeling (TUNEL) assay using blastocysts developed from the indicated groups. Merged images (light green) of TUNEL (green, white arrowheads) and DAPI (blue) signals are shown. (**l**) Quantification of the numbers and proportion of apoptotic cells in the indicated groups. Within each category, groups marked with different letters (a and b) are significantly different (*p* < 0.05). Bar = 100 μM.

Before IVM, COCs were classified as BCB- or BCB+, according to their coloration, following their exposure to BCB staining (Figure 1a,b) and subjection to IVM. After 44 h of IVM, the cumulus cell expansion and oocyte nuclear maturation of BCB- and BCB+ COCs were investigated. BCB+ COCs showed a significantly higher (*p* < 0.05) proportion of COCs exhibiting a complete cumulus cell expansion (degree 4, 79.6 ± 2.5% vs. 51.1 ± 1.9%) and lower (*p* < 0.05) proportions of degrees 1, 2, and 3 as compared with BCB- COCs (3.1 ± 0.3% vs. 10.4 ± 1.0%, 7.5 ± 1.4% vs. 18.6 ± 1.4%, and 9.8 ± 1.3% vs. 19.9 ± 1.3%, respectively) (Figure 1c,d). In terms of oocyte nuclear maturation (Figure 1e,f), the BCB+ group exhibited a significantly higher (*p* < 0.05) MII rate (95.2 ± 0.8% vs. 65.7 ± 2.3%) and lower (*p* < 0.05) degeneration and immature rates as compared with the BCB- group (0.7 ± 0.3% vs. 10.3 ± 1.5% and 4.0 ± 0.8% vs. 24.1 ± 1.9%, respectively).

### 2.2. Evaluation of the Subsequent Development of Parthenogenetic Embryos Derived from BCB- and BCB+ Oocytes

The subsequent development of PA embryos derived from BCB- and BCB+ oocytes was investigated. The BCB+ group showed a significantly higher (*p* < 0.05) cleavage and blastocyst formation rates after PA as compared with the BCB- group (95.8 ± 1.2% vs. 85.7 ± 1.6% and 52.6 ± 3.6% vs. 25.3 ± 2.6%, respectively) (Figure 1g,h). In addition, the BCB+ group showed a significantly higher (*p* < 0.05) total cell number (41.0 ± 0.9 vs. 33.4 ± 2.8), including the trophectoderm (TE) cell number (34.8 ± 0.9 vs. 27.7 ± 2.7) (Figure 1i,j), and a significantly lower (*p* < 0.05) number and percentage of apoptotic cells in blastocysts as compared with the BCB- group (1.7 ± 0.2 vs. 1.1 ± 0.1 and 4.9 ± 0.8% vs. 2.8 ± 0.1%, respectively) (Figure 1k,l).

### 2.3. Evaluation of the Subsequent Development of In Vitro Fertilized Embryos Derived from BCB- and BCB+ Oocytes

**Figure 2:**
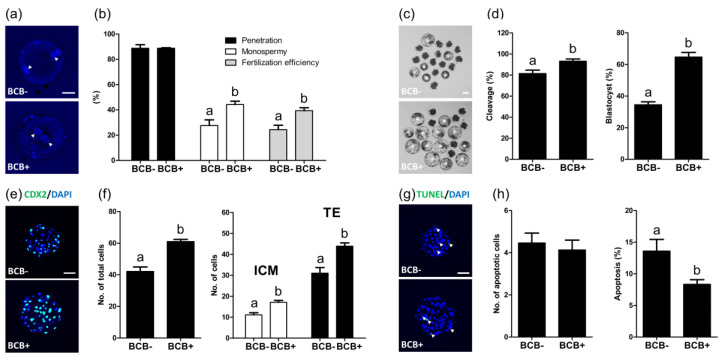
The subsequent development of in vitro fertilized embryos developed from BCB- and BCB+ porcine oocytes. (**a**) Representative photomicrographs of pronuclear stage zygotes developed from BCB- and BCB+ oocytes. (**b**) The penetration rate (percentage of penetrated oocytes/total inseminated oocytes), monospermy rate (percentage of oocytes containing only one male pronucleus/total oocytes penetrated), and fertilization efficiency (percentage of monospermic oocytes/total oocytes inseminated) of the zygotes developed from BCB- and BCB+ oocytes. (**c**) Representative photomicrographs of blastocysts (white asterisks), and (**d**) the cleavage and blastocyst formation rates of embryos developed from BCB- and BCB+ oocytes. (**e**) Immunocytochemistry of CDX2/4′,6-diamidino-2-phenylindole (DAPI) using blastocysts developed from the indicated groups. Merged images of CDX2 (green) and DAPI (blue) signals are shown. (**f**) Quantification of the total, inner cell mass (ICM), and trophectoderm (TE) cell numbers in the indicated groups. (**g**) Terminal deoxynucleotidyl transferase mediated dUTP digoxygenin nick end labeling (TUNEL) assay using blastocysts developed from the indicated groups. Merged images (light green) of TUNEL (green, white arrowheads) and DAPI (blue) signals are shown. (**h**) Quantification of numbers and proportion of apoptotic cells in the indicated groups. Within each category, groups marked with different letters are significantly different (*p* < 0.05). Bar = 100 μM.

The subsequent development of IVF embryos derived from BCB- and BCB+ oocytes was investigated. After 10 h of IVF, the penetration, monospermy, and fertilization efficiency were evaluated. While there was no difference in the penetration rate between the BCB- and BCB+ groups (88.7 ± 2.8% vs. 88.7 ± 0.3%), the BCB+ group exhibited a significantly higher (*p* < 0.05) monospermy and fertilization efficiency as compared with the BCB- group (44.3 ± 2.6% vs. 27.6 ± 4.5% and 39.3 ± 2.4% vs. 24.3± 3.5%, respectively) (Figure 2a,b). On day two and six after IVF, the cleavage and blastocyst formation rates were evaluated, and the BCB+ group showed significantly higher (*p* < 0.05) cleavage and blastocyst formation rates after IVF as compared with the BCB- group (93.1 ± 2.1% vs. 81.4 ± 3.2% and 64.7 ± 2.9% vs. 34.5 ± 3.7%, respectively) (Figure 2c,d). In addition, the BCB+ group showed a significantly higher (*p* < 0.05) total cell number (61.0 ± 1.4 vs. 42.1 ± 2.8), including the number of cells in the inner cell mass (ICM) and the number of TEs (17.1 ± 0.9 vs. 11.1 ± 1.0 and 43.9 ± 1.6 vs. 31.0 ± 2.7, respectively) (Figure 2e,f), and a significantly lower (*p* < 0.05) percentage of apoptotic cells in blastocysts as compared with the BCB- group (8.3 ± 0.7% vs. 13.6 ± 1.8%) (Figure 2g,h).

### 2.4. Evaluation of Apoptosis Levels in BCB- and BCB+ COCs

**Figure 3:**
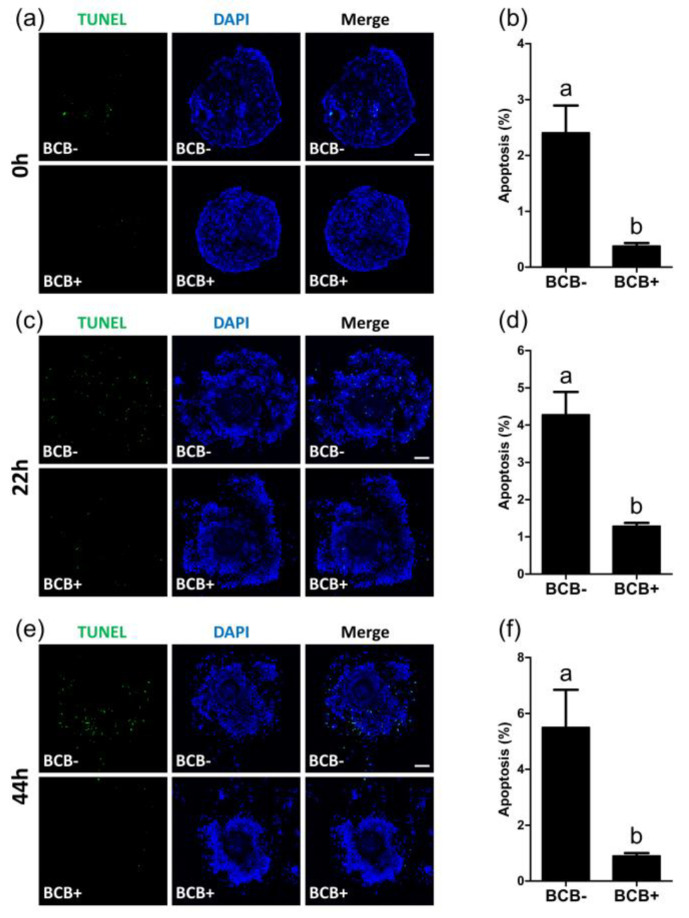
Detection of cellular apoptosis during the in vitro maturation of BCB- and BCB+ porcine cumulus-oocyte complexes (COCs), as detected by TUNEL staining. Representative TUNEL (green) assay images of COCs and quantification of TUNEL positive cells in BCB- and BCB+ COCs, after 0 h (**a**,**b**), 22 h (**c**,**d**), and 44 h (**e**,**f**) of in vitro maturation. Within each category, groups marked with different letters are significantly different (*p* < 0.05). Bar = 100 μM.

After 0, 22, and 44 h of IVM, the apoptosis levels in BCB- and BCB+ COCs were evaluated using a TUNEL assay. After 0 h of IVM, the BCB+ group showed a significantly lower (*p* < 0.05) percentage of apoptotic cells in COCs as compared with the BCB- group (0.4 ± 0.1% vs. 2.4 ± 0.5%) (Figure 3a,b). After 22 h of IVM, the BCB+ group exhibited a significantly lower (*p* < 0.05) percentage of apoptotic cells in COCs as compared with the BCB- group (1.3 ± 0.1% vs. 4.3 ± 0.6%) (Figure 3c,d). After 44 h of IVM, the BCB+ group showed a significantly lower (*p* < 0.05) percentage of apoptotic cells in COCs as compared with the BCB- group (0.9 ± 0.1% vs. 4.7 ± 1.0%) (Figure 3e,f).

### 2.5. Evaluation of the Expression of SHH-Signaling Proteins in the BCB- and BCB+ COCs

**Figure 4:**
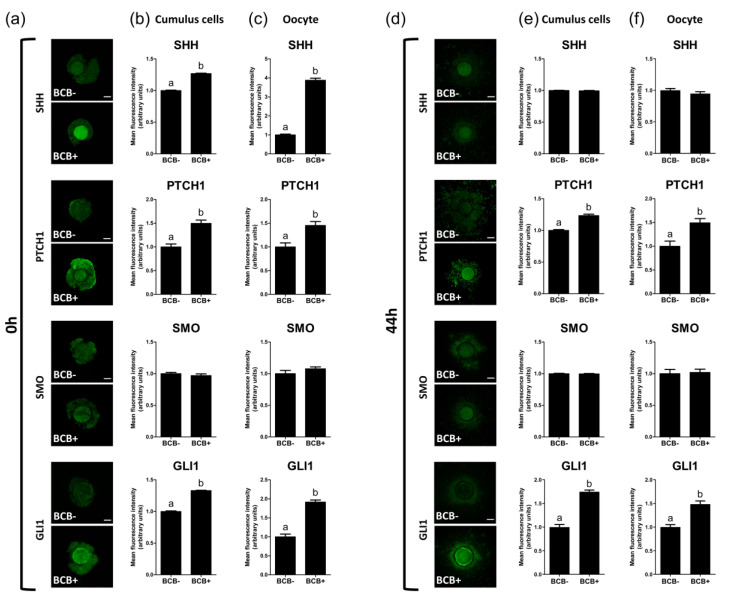
Immunocytochemical analysis of Sonic hedgehog (SHH)-signaling proteins in BCB- and BCB+ porcine cumulus-oocyte complexes (COCs). Representative images of porcine COCs of each group stained with antibodies directed against SHH, PTCH1, SMO, and GLI1 and comparison of expression in cumulus cells and oocytes after 0 h (**a**–**c**) and 44 h (**d**–**f**) of in vitro maturation, respectively. Within each category, groups marked with different letters are significantly different (*p* < 0.05). Bar = 100 μM.

The expression levels of SHH-signaling proteins were evaluated in BCB- and BCB+ COCs. After 0 h of IVM, BCB+ COCs showed significantly higher (*p* < 0.05) expression levels of SHH-signaling proteins, including SHH (cumulus cells, 1.3 ± 0.0 vs. 1.0 ± 0.0; and oocytes, 3.9 ± 0.1 vs. 1.0 ± 0.0), PTCH1 (cumulus cells, 1.5 ± 0.1 vs. 1.0 ± 0.1; and oocytes, 1.5 ± 0.1 vs. 1.0 ± 0.1), and GLI1 (cumulus cells, 1.3 ± 0.0 vs. 1.0 ± 0.0; and oocytes, 1.9 ± 0.1 vs. 1.0 ± 0.1) in both cumulus cells and oocytes as compared with the BCB- group (Figure 4a–c). After 44 h of IVM, BCB+ COCs exhibited significantly higher (*p* < 0.05) expression levels of SHH-signaling proteins, including PTCH1 (cumulus cells, 1.2 ± 0.0 vs. 1.0 ± 0.0; and oocytes, 1.5 ± 0.1 vs. 1.0 ± 0.1) and GLI1 (cumulus cells, 1.7 ± 0.0 vs. 1.0 ± 0.1; and oocytes, 1.5 ± 0.1 vs. 1.0 ± 0.1) in both cumulus cells and oocytes as compared with the BCB- group (Figure 4d–f).

## 3. Discussion

In the current study, we demonstrated that high-quality oocytes, assessed by BCB staining, have a greater potential to expand their surrounding cumulus cells with active SHH signaling and a lower apoptosis. Following IVM, BCB+ COCs showed a significantly higher cumulus cell expansion and oocyte nuclear maturation as compared with BCB- COCs. Furthermore, BCB+ COCs exhibited lower apoptosis levels and a higher expression of SHH-signaling proteins as compared with BCB- COCs. After PA and IVF, the BCB+ oocytes showed a higher developmental competence than the BCB- oocytes. These results suggested that high-quality COCs assessed by BCB staining showed a high potential to expand when surrounding cumulus cells with active SHH signaling and a lower apoptosis.

Recently, BCB staining has been successfully applied to select high-quality oocytes for IVM in various species [7]. To determine safe conditions for this dye, various concentrations and incubation times have been tested. A concentration of 26 µM and incubation time of 90 min were found to be safe and effective for pig, cow, goat, and mouse oocytes, as it was supported by a high rate of development, without an apparent loss of viability [23]. In addition, it has been consistently reported that BCB+ oocytes showed a higher subsequent embryo development and quality of blastocysts as compared with BCB- oocytes in several species, such as pigs [24], cattle [9], and sheep [25]. In pigs, Pawlak et al. [26] demonstrated that the exposure of oocytes derived from prepubertal gilts to a BCB solution could affect the rate of the first polar body extrusion and the incidence of chromosomal abnormality as compared with a control group (that was not subjected to BCB staining). In addition, in another study by Pawlak et al. [27], there was no difference in cytoplasmic maturation between the BCB+ and control oocytes. This could be because these phenomena were related to the extra 90 min of incubation in the BCB solution, or because most of the control oocytes consisted of BCB+ oocytes. However, in the present study, because we compared BCB- and BCB+ COCs in terms of several parameters, exposing them to a BCB solution under the same conditions (26 μM of BCB for 90 min), the effect of BCB staining itself on the results could be excluded. We used BCB staining as a reliable tool to differentiate oocyte quality (BCB- as low-quality and BCB+ as high-quality) and to investigate what drives the higher developmental competence of BCB+ oocytes during IVM.

Taking the in vivo physiology of oocyte maturation into account, the higher developmental competence of high-quality oocytes could be closely related to cumulus cell expansion. In growing ovarian follicles, communication between the oocyte and surrounding somatic cells regulates their proliferation and differentiation [20]. Specifically, oocyte and cumulus cells communicate with each other by cell–cell communication via gap junctions [28]. Therefore, by affecting each other’s development, intercellular interactions between the oocyte and cumulus cells are considered to play an important role in oocyte maturation [29]. According to the physiological conditions, it was assumed that different levels of cumulus cell expansion would be observed between high- and low-quality COCs after maturation. As expected from previous studies [4,9,24], a higher developmental competence of BCB+ oocytes was observed in the present study, which was supported by higher MII rates and a subsequent embryo development after PA and IVF in the BCB+ group as compared with the BCB- group. In addition, the BCB+ group showed a significantly higher proportion of complete cumulus cell expansion (degree 4), and therefore a significantly lower proportion of incomplete cumulus expansion (degree 1, 2, and 3) as compared with the BCB- group, indicating that high-quality oocytes had a greater potential to expand their surrounding cumulus cells. Furthermore, these biological processes could be regulated via signaling pathways, such as SHH signaling, which controls follicle development [19]. Recently, targets of active SHH signaling (e.g., PTCH, SMO, and GLI1) were identified in the granulosa and cumulus cell layers of the ovaries in various species, including mice [30], humans [30], goats [31], cattle [32], and pigs [22]. Although in goats, transcripts of *SHH*, *PTCH1*, *SMO*, and *GLI1* were expressed in the ovaries, granulosa cells, cumulus cells, oocytes, and oviduct epithelia except for the expression of *PTCH1* in cumulus cells [31], all of these active SHH signaling targets were expressed in the granulosa cells, cumulus cells, and oocytes in pigs [22]. Therefore, we hypothesized that high-quality oocytes could affect one of the signaling pathways that drive the expansion of cumulus cells in vitro. In the present study, the BCB+ group showed significantly higher expression levels of SHH-signaling proteins in both cumulus cells and oocytes at 0 h (SHH, PTCH1, and GLI1) and 44 h of IVM (PTCH1 and GLI1), indicating that high-quality COCs selected by BCB staining had a higher expression of SHH-signaling proteins. This result suggested that a higher expression of SHH-signaling proteins during the maturation of COCs could be related to higher cumulus cell expansion, oocyte nuclear maturation, and subsequent embryo development which were shown in high-quality COCs. In particular, a significantly higher SHH expression in the BCB+ group was only observed at 0 h of IVM. Considering the evidence that the highest level of SHH protein was observed in the follicular fluid of small follicles, where granulosa and cumulus cells proliferate actively [22], it is reasonable to speculate that a high SHH expression at the beginning of IVM could be required for a proper cumulus cell expansion. In addition, the expression of SMO in the BCB+ and BCB- groups was not significantly different, both before and after IVM. This could be because the regulation of SMO levels does not depend on the transcription factor of Hedgehog signaling but occurs post-transcriptionally [33], whereas the regulation of PTCH1 and GLI1 levels depends on transcription mediated through Hedgehog signaling [34,35]. On the basis of these results, we concluded that high-quality COCs showed a higher cumulus cell expansion with a higher expression of SHH-signaling proteins.

In terms of the relationship between oocyte quality and cumulus cell apoptosis, it has been previously reported that oocytes prevent cumulus cell apoptosis by maintaining a morphogenic paracrine gradient of oocyte-secreted factors [36]. Furthermore, it has been demonstrated that apoptosis in cumulus cells, but not in oocytes, was correlated with oocyte developmental competence [37]. In the present study, the BCB+ group exhibited significantly lower apoptosis levels in cumulus cells and a higher oocyte nuclear maturation and subsequent embryo development after PA and IVF, indicating that high-quality oocytes reduced apoptosis in cumulus cells, thereby leading to a better subsequent embryo development. Taken together, the higher developmental competence of BCB+ oocytes could be explained by their higher potential to expand cumulus cells with active SHH signaling and reduced rates of apoptosis.

In conclusion, oocyte quality, assessed by BCB staining, significantly affected the cumulus cell expansion, SHH signaling, and apoptosis levels in COCs. Considering the interactive cellular milieu among the oocyte and surrounding cumulus cells, a higher cumulus cell expansion with active SHH signaling and a reduced apoptosis in high-quality COCs could provide a proper environment for oocyte maturation, thereby leading to a better subsequent embryo development. These findings are useful for providing insights into the role of Sonic hedgehog signaling in the maturation of COCs. However, further studies are required to elucidate the complete process by which oocyte quality affects cumulus cell expansion and SHH signaling during the maturation of COCs using the SHH protein or SHH inhibitor.

## 4. Materials and Methods

### 4.1. Chemicals

All chemicals and reagents used in this study were purchased from Sigma-Aldrich Chemical Company (St. Louis, MO, USA), unless otherwise stated.

### 4.2. Isolation of COCs

Porcine ovaries were collected from prepubertal gilts at a local abattoir and transported to the laboratory in physiological saline at 32–35 °C. The COCs were aspirated from superficial ovarian follicles (3–6 mm diameter) using a disposable 10 mL syringe with an 18-gauge needle and allowed to settle as sediment in 50 mL conical tubes at 37 °C for 5 min. The supernatant was removed, and the sediment was washed three times with Tyrode’s Albumin Lactate Pyruvate-HEPES medium. Only COCs with at least three uniform layers of compact cumulus cells and a homogeneous cytoplasm were used for the experiments.

### 4.3. Brilliant Cresyl Blue Staining

Immediately after the COCs were isolated, they were incubated in a Dulbecco’s phosphate-buffered saline (DPBS; Gibco, Grand Island, NY, USA) supplemented with a 0.4% bovine serum albumin (BSA) (PB1) medium containing 26 μM of BCB for 90 min at 38.5 °C in humidified air. After incubation, the COCs were washed three times in a PB1 medium and classified into the following two groups, depending on the coloration of the cytoplasm: oocytes without blue coloration (BCB- group) and oocytes with blue coloration (BCB+ group). After classification, the COCs were washed twice in an IVM medium and subjected to IVM.

### 4.4. In Vitro Maturation of Oocytes

The IVM medium was composed of a tissue culture medium (TCM)-199 supplemented with 10% porcine follicular fluid, 10 ng/mL of β-mercaptoethanol, 0.57 mM of cysteine, 10 ng/mL of epidermal growth factor, 10 IU/mL of pregnant mare serum gonadotropin (PMSG; Prospec Bio, East Brunswick, NJ, USA), and 10 IU/mL of human chorionic gonadotropin (hCG; Prospec Bio). Approximately 50 COCs were matured in 500 μL of the IVM medium in a four-well multi-dish (Nunc, Roskilde, Denmark) at 38.5 °C with 5% CO_2_ in 95% humidified air. After 21–22 h of maturing the culture with hormones, the COCs were washed twice in a fresh hormone-free IVM medium and cultured in a hormone-free IVM medium for an additional 21–22 h.

### 4.5. Assessment of Cumulus Cell Expansion

A total of 1063 COCs was used in seven independent replicates. After 44 h of IVM, the degree of cumulus cell expansion was assessed by microscopic examination, as previously described [38]. Briefly, a degree of 0 indicates no expansion, which is characterized by a detachment of cumulus cells from the oocyte, leaving a partially or fully denuded oocyte. A degree of 1 indicates the minimum observable response, with spherical and compacted cumulus cells around the oocyte. A degree of 2 indicates that only the outermost layers of the cumulus cells have expanded. A degree of 3 indicates that all cell layers, except for the corona radiate, have expanded. A degree of 4 indicates the maximum degree of expansion of the cell layers, including the corona radiata.

### 4.6. Assessment of the Nuclear Maturation of Oocytes

A total of 1043 oocytes was used in seven independent replicates. After 44 h of IVM, the COCs were denuded by gently pipetting with 0.1% hyaluronidase in a PB1 medium and washed three times in a PB1 medium. The denuded oocytes were evaluated under a microscope (Nikon Corp., Tokyo, Japan) and classified as immature (without a first polar body extrusion), degenerate (with a broken oolemma or abnormal looking cytoplasm), or metaphase II (MII) (with a first polar body extrusion) [11].

### 4.7. Parthenogenetic Activation of Oocytes

A total of 509 oocytes was used in five independent replicates. For PA, the COCs were denuded by gently pipetting with 0.1% hyaluronidase, after 44 h of IVM culturing, washed three times in a PB1 medium, and gradually equilibrated in an activation medium consisting of 0.28 M of mannitol, 0.5 mM of HEPES, 0.1 mM of CaCl_2_·2H_2_O, 0.1 mM of MgSO_4_·7H_2_O, and 0.01% polyvinyl alcohol (PVA). For activation, the MII oocytes were placed between electrodes covered with an activation medium in a chamber connected to an Electro Cell Fusion Generator (LF 101; Nepa Gene, Chiba, Japan). The oocytes were activated with a single direct current (DC) pulse of 1.1 kV/cm for 50 μs. The electrically-activated oocytes were cultured in a porcine zygote medium-3 (PZM-3) supplemented with 2 mM of 6-dimethylaminopurine and 5 μg/mL of cytochalasin B for 4 h at 38.5 °C in a humidified atmosphere of 5% CO_2_. After 4 h, the activated oocytes were washed and cultured in PZM-3 at 38.5 °C in 5% CO_2_ in air for 6 days.

### 4.8. In Vitro Fertilization of Oocytes

A total of 181 oocytes was used in four independent replicates to evaluate the embryo development after IVF. A total of 108 oocytes was used in three independent replicates to evaluate the number of pronuclei. For IVF, the COCs, after 44 h of IVM culturing, were denuded by gently pipetting with 0.1% hyaluronidase in a PB1 medium. Then, they were washed three times in a modified Tris-buffered medium (mTBM) containing 2.5 mM of caffeine sodium benzoate and 1 mg/mL of BSA. Next, 10–15 oocytes were placed into 48 µL droplets of an IVF medium under mineral oil pre-equilibrated at 38.5 °C in 5% CO_2_ in air. To prepare the spermatozoa using the swim-up method before fertilization, the semen sample was kept at 17 °C for 1–3 days and washed three times with a sperm washing medium (DPBS (Gibco) supplemented with 1 mg/mL of BSA, 100 µg/mL of penicillin G, and 75 µg/mL of streptomycin sulfate). After washing, 2 mL of the sperm washing medium was gently added to the spermatozoa pellet and incubated for 15 min at 38.5 °C in 5% CO_2_ in air. After incubation, the supernatant was washed with mTBM and resuspended with 1 mL of mTBM. The initial sperm concentration of the supernatant was determined by computer-assisted sperm analysis (CASA) and diluted in order to obtain 3.75 × 10^6^ spermatozoa/mL. Then, 2 µL of diluted spermatozoa was added to a 48 µL droplet of mTBM containing 10–15 oocytes, giving a final concentration of 1.5 × 10^5^ spermatozoa/mL. The oocytes were co-incubated with the spermatozoa for 6 h at 38.5 °C in 5% CO_2_ in air. After 6 h, oocytes were stripped by gentle pipetting and transferred to PZM-3 for culturing at 38.5 °C in 5% CO_2_ in air. To count the pronuclei, fertilized embryos, at 10 h after insemination, were washed three times with DPBS supplemented with 0.1% PVA (DPBS-PVA) and fixed with 4% paraformaldehyde overnight at 4 °C. Then, they were washed three times in DPBS-PVA and mounted on glass slides with 2 µg/mL of a Vectashield mounting medium using 4′,6-diamidino-2-phenylindole (DAPI) (Vector Laboratories, Burlingame, CA, USA). The pronuclei were observed under UV light using an epifluorescence microscope (Leica DMi8; Leica Microsystems, Wetzlar, Germany).

### 4.9. Terminal Deoxynucleotidyl Transferase Mediated dUTP Digoxygenin Nick End Labeling (TUNEL) Assay 

In total, 24, 42, and 28 COCs were sampled at different time points (0, 22, and 44 h of IVM, respectively) and used in three independent replicates. A total of 18 PA-derived and 30 IVF-derived blastocysts was used in four independent replicates. To evaluate the apoptotic cells in COCs or blastocysts, a TUNEL assay was performed using an in situ cell death detection kit (Roche, Basel, Switzerland). The COCs or blastocysts were washed three times in DPBS-PVA and fixed in 4% paraformaldehyde overnight at 4 °C. Fixed COCs or blastocysts were permeabilized in DPBS containing 1% Triton X-100 at room temperature for 60 min. Subsequently, COCs or blastocysts were washed three times with DPBS-PVA and incubated with fluorescein-conjugated dUTP and terminal deoxynucleotidyl transferase for 1 h at 38.5 °C. After incubation, the COCs or blastocysts were washed three times with DPBS-PVA and mounted on clean glass slides with 2 µg/mL of Vectashield mounting medium using DAPI (Vector Laboratories). DAPI-labeled or TUNEL-positive nuclei were observed under a fluorescence microscope. COCs were observed under a laser-scanning confocal fluorescence microscope (LSM700; Zeiss, Oberkochen, Germany). Blastocysts were observed under a fluorescence microscope (Leica DMi8; Leica Microsystems). Apoptotic cell numbers per COC or blastocyst were judged by counting the nuclei with blue (DAPI) and green (TUNEL) signals. To calculate the percentage of apoptosis in COCs, TUNEL-positive and DAPI-positive cumulus cells were counted using the ImageJ software (National Institutes of Health, MD, USA), and the percentage of TUNEL-positive cells, relative to the total number of cumulus cells (DAPI-positive) in the same COC, was calculated.

### 4.10. Immunocytochemical Staining

For SHH staining in COCs, in total, 34 and 32 COCs were sampled at different time points (0 and 44 h of IVM, respectively) and used in three independent replicates. For PTCH1 staining in COCs, in total, 30 and 22 COCs were sampled at different time points (0 and 44 h of IVM, respectively) and used in three independent replicates. For SMO staining in COCs, in total, 40 and 24 COCs were sampled at different time points (0 and 44 h of IVM, respectively) and used in three independent replicates. For GLI1 staining in COCs, in total, 30 and 34 COCs were sampled at different time points (0 and 44 h of IVM, respectively) and used in three independent replicates. For Caudal-type homeobox 2 (CDX2) staining in blastocysts, a total of 18 PA-derived and 20 IVF-derived blastocysts was used in four independent replicates. The COCs or blastocysts were fixed in 4% paraformaldehyde overnight at 4 °C and washed three times in DPBS-PVA for 10 min each. For membrane permeabilization, the fixed COCs or blastocysts were incubated in DPBS containing 1% Triton X-100 for 1 h at room temperature. Subsequently, the COCs or blastocysts were washed three times in DPBS-PVA and stored in DPBS-PVA supplemented with 1% BSA (DPBS-PVA-BSA) at 4 °C overnight for blocking. To stain the SHH-signaling proteins in COCs, the COCs were incubated overnight at 4 °C with mouse monoclonal primary antibodies against SHH (1:200; sc-365112; Santa Cruz Biotechnology Inc., Dallas, TX, USA), PTCH1 (1:200; sc-293416; Santa Cruz Biotechnology Inc.), SMO (1:200; sc-166685; Santa Cruz Biotechnology Inc.), and GLI1 (1:200; sc-515751; Santa Cruz Biotechnology Inc.). For CDX2 staining, blastocysts were additionally blocked with 10% normal goat serum for 1 h. Next, blastocysts were incubated overnight at 4 °C with mouse monoclonal primary antibody against CDX2 (an undiluted solution; AM392; Biogenex Laboratories Inc., San Ramon, CA, USA). Subsequently, the COCs or blastocysts were washed three times in DPBS-PVA-BSA for 10 min each and incubated for 1 h at room temperature with conjugated secondary antibody, Alexa Fluor 488-labeled goat anti-mouse IgG (1:200 in DPBS-PVA-BSA). After the COCs or blastocysts were washed three times in DPBS-PVA-BSA for 10 min each, they were mounted with 2 µg/mL of the Vectashield mounting medium using DAPI (Vector Laboratories) on clean glass slides. COCs were observed under a laser scanning confocal fluorescent microscope (Zeiss LSM700; Zeiss). Blastocysts were observed under a fluorescence microscope (Leica DMi8; Leica Microsystems).

### 4.11. Statistical Analysis

Statistical analyses were performed using Sigmastat software (SPSS, Inc., Chicago, IL, USA). All data were tested for normality and homoscedasticity, and then subjected to a Mann–Whitney *U*-test for data with a non-normal distribution (Figure 1I left panel, Figure 2f right panel and 2h left panel, Figure 3f, Figure 4b SHH, PTCH1, and SMO, Figure 4c SMO, and Figure 4e SHH, SMO, and GLI1) or a Student’s *t*-test for data with a normal distribution (all data except the data compared by the Mann–Whitney *U*-test) to determine the differences between the experimental groups. Data are expressed as the mean ± standard error of the mean. Differences at *p* < 0.05 were considered statistically significant.

## Abbreviations

IVM	In vitro maturation
ART	Assisted reproductive technology
BCB	Brilliant cresyl blue
G6PDH	Glucose-6-phosphate dehydrogenase
SHH	Sonic hedgehog
HA	Hyaluronic acid
PTCH1	Patched 1
SMO	Smoothened
GLI1	Glioma-associated oncogene homolog 1
COCs	Cumulus-oocyte complexes
PA	Parthenogenetic activation
IVF	In vitro fertilization
TE	Trophectoderm
DPBS	Dulbecco’s phosphate-buffered saline
BSA	Bovine serum albumin
TCM	Tissue culture medium
PMSG	Pregnant mare serum gonadotropin
hCG	Human chorionic gonadotropin
MII	Metaphase II
PVA	Polyvinyl alcohol
PZM-3	Porcine zygote medium-3
mTBM	Modified Tris-buffered medium
CASA	Computer-assisted sperm analysis
DAPI	4′,6-diamidino-2-phenylindole
TUNEL	Terminal deoxynucleotidyl transferase mediated dUTP digoxygenin nick end labeling
CDX2	Caudal type homeobox 2
